# Rethinking brain injury after subarachnoid hemorrhage

**DOI:** 10.1186/s13054-020-03342-2

**Published:** 2020-10-17

**Authors:** Sara Amodio, Pierre Bouzat, Chiara Robba, Fabio Silvio Taccone

**Affiliations:** 1 Dipartimento di Scienze Chirurgiche e Diagnostiche Integrate, University of Genova, Genova, Italy; 2 Department of Anesthesiology and Intensive Care, University of Grenoble, Grenoble, France; 3 Policlinico San Martino, IRCCS for Oncology and Neuroscience, Genova, Italy; 4 Department of Intensive Care Medicine, Erasme Hospital, Université Libre de Bruxelles (ULB), Route de Lennik, 808, 1070 Brussels, Belgium

## Introduction

Aneurysmal subarachnoid hemorrhage (aSAH) has an annual incidence of six cases per 100,000 persons, with a high incidence of long-term poor neurological outcome [1, 2]. Several complications may occur after the aneurysm rupture, such as global cerebral ischemia, re-bleeding, medical complications (i.e., infections, anemia and hyponatremia) and the occurrence of secondary brain injury [2]. As such, poor outcome is strongly related to the occurrence of initial (i.e., early brain injury) or delayed events (i.e., delayed cerebral ischemia). However, the nomenclature of these events and their diagnosis/management need to be better specified.

## Early brain injury

The concept of “early brain injury” (EBI) after SAH has been recently introduced and encompasses several disorders occurring within the first 72 h following the aneurysm rupture [3]. Definition of EBI is heterogeneous, as mainly based on experimental data, and includes the evaluation of initial clinical symptoms, neuroimaging findings, as well as metabolic and/or electrophysiological variables using multimodal neuromonitoring (i.e., cortical spreading depolarizations [CSDs], which are detected by intracerebral electroencephalography, iEEG; tissue hypoxia; metabolic distress, which is detected by cerebral microdialysis, cMD) [3]. EBI results primarily from extravasation of blood into the subarachnoid space and increased intracranial pressure (ICP), which result in reduced cerebral blood flow (CBF) and transient global cerebral ischemia [4, 5]. These processes could be further aggravated by cerebral vasoconstriction, disruption of the brain blood barrier (BBB), neuro-inflammation, early seizures or loss of CBF autoregulation [6, 7], which will further contribute to increase ICP and brain ischemia. Although not proven in large randomized clinical trials, several interventions might be initiated (i.e., modulation of MAP, osmotic therapies, anticonvulsive therapy and normothermia) to minimize the extent of EBI in these patients [8].

## Delayed cerebral ischemia: towards a new definition?

Regardless of the initial clinical presentation, aSAH patients may present a delayed neurological deterioration. In 2010, a definition of “clinical deterioration caused by delayed cerebral ischemia” (DCI) was proposed, as “*the occurrence of focal neurological impairment, such as hemiparesis, aphasia, apraxia, hemianopsia, or neglect, or a decrease of at least 2 points on the Glasgow Coma Scale…*” [9]. However, this definition presents several limitations. First, it does not take into account other more subtle clinical signs, such as those related to frontal (mood changes) or posterior cerebral areas (i.e., dysgraphia and dyscalculia). Second, this definition is of limited use in unconscious or sedated patients, in whom clinical assessment is unreliable. Third, the presence of an already established cerebral ischemia on neuroimaging would not allow physicians to initiate adequate therapies to avoid tissue hypoxia. Forth, the definition of clinical deterioration after DCI does not provide information on the underlying mechanisms; even though cerebral vasospasm has been considered for decades as the main determinant of DCI [10], other pathological phenomena could be involved [11], including also systemic medical complications [12].

**Fig. 1:**
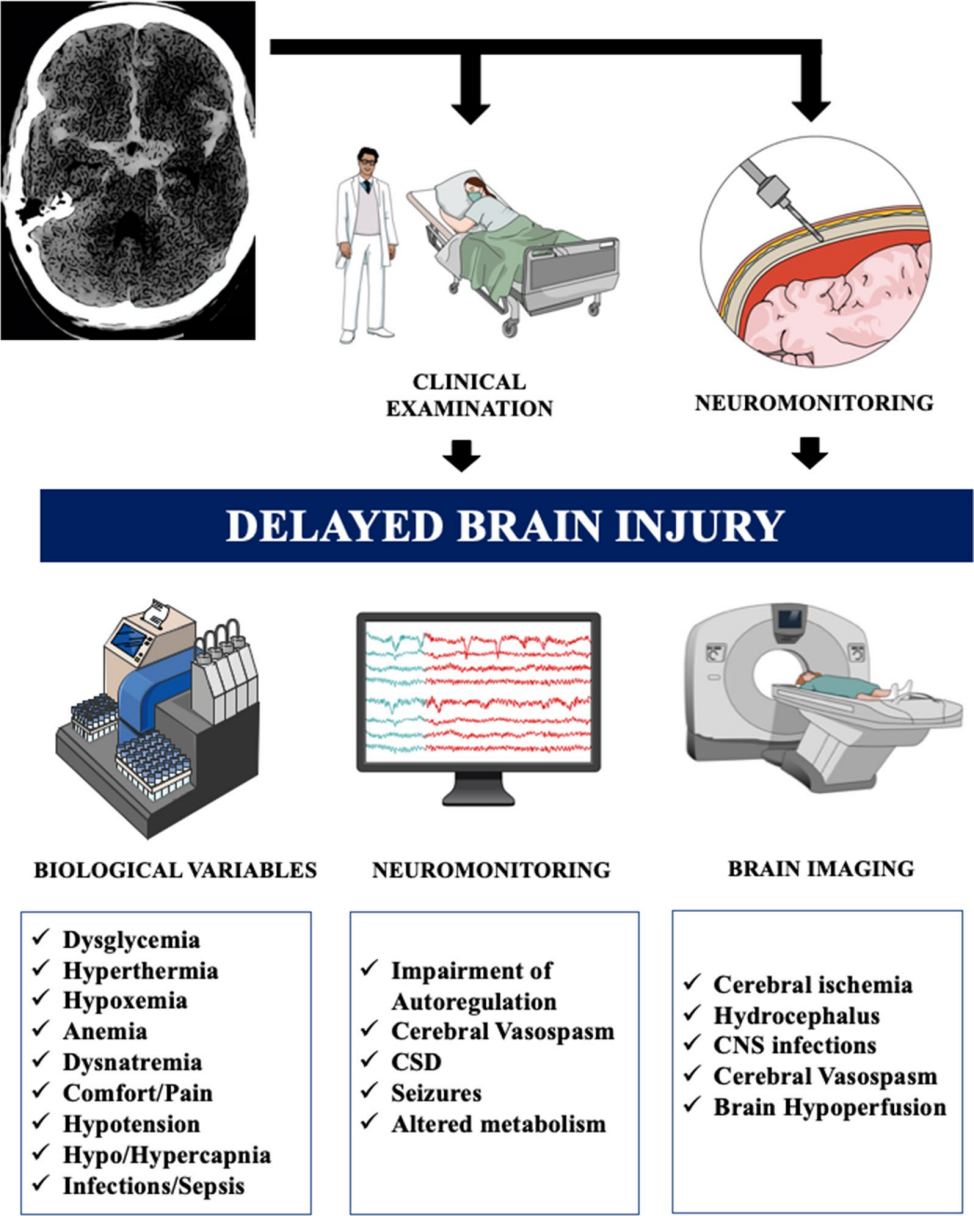
Practical approach to the identification of delayed brain injury after subarachnoid hemorrhage and some of the potential etiologies, according to different diagnostic approaches

Therefore, as the pathophysiology of delayed neuro-worsening of SAH patients includes also “non-ischemic” processes, secondary brain injury should be more specifically defined as a “Delayed Brain Injury” (DBI). As such, DBI diagnosis should be based on repeated clinical examinations and/or neuro-monitoring tools (i.e., altered brain hemodynamics, oxygenation or metabolism—Fig. 1) [13]. When delayed brain injury is suspected, biological and microbiological tests as well as systemic monitoring should be used to rule out different causes of DBI, either cerebral (i.e., delayed hydrocephalus, arachnoiditis, seizures, ventriculitis, loss of autoregulation and/or cerebral vasospasm and cerebral thromboembolic events) or systemic (i.e., dysglycemia, electrolytic derangements, cardiovascular and respiratory failure or sepsis). As such, multimodal monitoring, such as direct measuring of intracranial pressure (ICP), brain tissue oxygen evaluation (PbtO_2_) and cerebral microdialysis (cMD), together with continuous EEG monitoring (cEEG), transcranial Doppler (TCD) and near-infrared spectroscopy (NIRS) are available real-time tools that might adequately characterize the cerebral etiologies of DBI, help to target therapy and to quantify brain response to therapeutic interventions [14].

Importantly, as different neuromonitoring tool provide non-redundant and complementary information, the use of a multimodal approach could further improve the understanding of the pathophysiology of DBI in this setting. As an example, TCD could early detect the alterations of cerebral blood flow velocities, while NIRS and PbtO_2_ may effectively assess regional oxygenation, either noninvasively or invasively. The occurrence of seizures can be adequately identified by the use of cEEG, while cMD would help to diagnose altered neuronal metabolism [15]. Although potentially useful, cerebral blood flow and autoregulation assessment have a limited role for routine bedside. Finally, cerebral computed tomography (CT), together with CT-angiography (CTA) and perfusion (CTP) are accurate tools to provide a definitive diagnosis of cerebral hypoperfusion [2] and microvascular dysfunction.

Once the diagnosis is made, several therapeutic options could be considered, according to the underlying cause, which may include, among all, induced hypertension, intra-arterial vasodilators, intracranial angioplasty for cerebral vasospasm, electrolytes replacement, optimization of systemic oxygen and hemoglobin, as well as antiepileptic therapy or surgical treatment for hydrocephalus. However, for microvascular dysfunction and cortical depolarization as causes of secondary brain injuries after SAH, no standardized therapies have been reported yet.

In summary, the concept of DBI should be implemented in the current management of SAH diagnosis and management. The advantages of this new approach are:

- The use of a more comprehensive term including different etiologies of delayed neurological deterioration and a more comprehensive description of the pathophysiology underlying this phenomenon;
- A more careful attention to non-ischemic and systemic processes which can lead to secondary brain injury and, eventually, to poor outcome;
- The need of a standardized multimodal neuromonitoring approach, using both invasive and noninvasive tools, to assess cerebral causes of DBI following SAH.

Further research is warranted to assess whether this new definition of pathological events occurring after the aneurysm rupture can favor a more precise characterization of therapeutic strategies aiming to improve patients’ management and outcome in this setting.

## Data Availability

Not Applicable.
